# Abstract of a Report on Dental Anesthesia

**Published:** 1905-03-15

**Authors:** E. Sauvez

**Affiliations:** Paris, France


### ABSTRACT OF A REPORT ON DENTAL ANESTHESIA.

BY DR. E. SAUVEZ, PARIS, FRANCE.

Presented to the International Dental Congress.

Struck with the exclusive use of general anesthesia by our American confreres, we have thought to engage their interest by exposing the benefits they might derive from our experience in twelve years of practice.

In 1893, we wrote a thesis on "The Best Method of Anesthesia to Employ in Dentistry." We extolled the use of bromid of ethyl for complete anesthesia. Two years of experiments had not then eradicated a certain fear concerning the use of cocain. Since then, statistics covering nearly fifteen thousand cases of injections of cocain with neither accident nor incident, have made us a warm partisan of local anesthesia.

We should like therefore to plead in favor of a local anesthesia in which we have absolute confidence, thoroughly persuaded that we speak in the interests of the patients, and of the dentists, and feeling assured that operations will be thereby better executed.

Without referring to the inconveniences in the use of all general anesthetics—examination of the heart and lungs, the function of the liver, the presence of one or more acids—we arrive at the main objection to the employment of general anesthesia, the possibility of death.

General anesthetics act by inducing functional arrest in the nervous centers, according to an immutable sequence; in the first place in the tegument, then in the medular, reflex centers, and finally in the bulb; if anesthesia is still further developed, the bulbous centers become paralyzed and death ensues.

According to the Edinburg *Medical Journal*, of November, 1903, the number of mortal cases produced by general anesthesia are divided as follows:

| | |
|---|---|
| Chlorid of Ethyl | 1—16.000 |
| Bromid of Ethyl | 1— 4.000 |
| Ether | 1—12.000 |
| Chloroform | 1—. 2.000 |

We do not mention the frequent and serious accidents, the blue syncopes from which the patients are only saved by artificial respiration. According to Prof. Reclus, "For an accident to count, it must be fatal."

In case of fatal accident, when the matter is brought to court, the judges, who would never question the use of an anesthetic for a serious operation, will often bitterly contest its employment when it is merely the question of the extraction of a tooth.

The results of general anesthesia should be noted, the cephalalgies, the nausea, lumbago, the peculiar state of illness, and the insomnia which may last for several consecutive days. With anesthesia of short duration by somnoform or nitrous oxid, a hasty operation becomes necessary and mistakes and accidents may result. Decided advantages should be attributed to complete anesthesia; among others, the patient does not see himself operated upon, several extractions can be made in one seance, and, according to some authors, cicatrization takes place more rapidly. Of three arguments, we believe that the first two in no wise balance the inconveniences and dangers of which we have spoken, and the last one is at least open to argument. Reclus, who employes cocain for general surgery, has never observed that cicatrization by first intention was any slower by this method, than in the case of general anesthesia.

If we consider the dangers and inconveniences of local anesthesia, we can affirm a clear record, as no mortal case has been observed under the use of twenty-three drops of a one percent solution of cocain, which is more than sufficient for average dental operations.

The risk of illness at most is only a slight vertigo, of insignificant proportions, and only with patients predisposed thereto, at any rate, never serious.

In two cases extracting wisdom teeth with trismus, local anesthesia was contra-indicated.

In other cases of acute arthritis and abscess, local anesthesia merely determined attenuation of the pain. Among the numerous advantages presented by cocain anesthesia we lay stress notably on time, tranquility, and the several facilities offered by cocain to the operator to accomplish surgical intervention with leisure and steadiness.

Many substances recommended for local anesthesia we have abandoned as impracticable; for instance, tincture of cannabis indica, strong carbolic acid, orthoform.

Electricity has been employed in two ways: First, by applying a current of high frequence directed toward the level of a tooth during a variable period previous to extraction.

Second, by the introduction of medical substances within the tissues, by the aid of electricity. This is the cataphoreses based on the theory of Farada's ions.

We need only to call attention to the complicated apparatus needed for administering these agents to show that electricity, as an agent of anesthesia, is not to be depended upon, presenting many inconveniences and difficulties.

We are thus forced to bring forward cocain, or its derivatives, which, in injections, seem to give practical results superior to all other methods of local anesthesia known at present.

Practically, cocain is only a local anesthetic; but, from a physiological standpoint, it is a general as well as a local anesthetic. The complete anesthesia attributed to cocain has been much discussed. The research on this subject by Mosso and Albertoni have caused cocain to be recognized as possessing the characteristics of a general anesthetic, because of its universal and temporary action on the cells. Charpentier de Manez assures us that this is a true characteristic of cocain, as far as germination and fermentation are concerned. Injection into animals in physiological doses, cocain determines in them an extreme muscular excitation, followed by an insensibility exclusively of the surface, the deeper sensibility being preserved.

It is this fact that caused Laffont and Arloing to consider cocain as a sensitive curare which acted exclusively on the sensitive extremities, just as this last alkaloid affects the motor extremities. This theory of a sensitive curare is combated by Mosso; an injection of cocain in the body of a nerve brings about anesthesia of the adjacent territory dependent on that nerve. Which fact induced Feinberg, Oberst, Pernice, Mans and Reclus to create regional anesthesia, which furthermore permitted the conception and execution of rachi-cocainization (Bier), in which the action of the cocain is directed to the rachidian roots.

One of the most remarkable of the properties of cocain is its vaso-constrictive action. We emphasize this fact and cite its divers consequences. Let us here only cite the elevation of the blood pressure. We would dwell on its action on the centers of thermic regulation (cocian heightens the temperature, Richet), and on the ocular apparatus.

We would call attention to the dangers which the use of cocain might offer. In the first place, there is syncope. We cite its rarity and how easy it is to avoid it by employing the right dose on the reclining patient. The other phenomena observed, sometimes subsequent even to correct injections of cocain, are slight and insignificant; slight tingling of the extremities and greater loquacity. There is really nothing in local anesthesia by cocain approaching the sudden scares which accompany the use of chloroform. Statistics bear us out in this statement. In 7,000 cases of anesthesia by cocain practiced by Reclus, he has not noted the slightest trouble in the physiological equilibrium. With a record of 15,000 injections we can not register one accident, not even an incident, due to the use of cocain. We are therefore in a position to affirm that this is the most inoffensive of all the anesthetics and that it exposes one to no surprise whatsoever.

With the idea of avoiding the so-called inconveniences ttributed to cocain, we have experimented with derivatives or congenerate substances. We will here limit ourselves with citing the conclusions we arrived at concerning tropa-cocain and eucain, etc.

Tropa-cocain presents an equal degree of toxicity while its anesthetic action appears less profound than that of cocain.

Eucain is a vaso-dilator, its injection is painful and it presents a feebler power of anesthesia and of a shorter period of duration than cocain.

Phenate of cocain, insoluble in water, and employed therefore, dissolved in oil or liquid vaseline, produces nodules which are long in disappearing.

We conclude, therefore, that the use of hydrochlorate of cocain appears to us to carry with it all the advantages claimed for the preparations compared to it as much from the point of view of the anesthesia produced as by the percentage of possible accidents.

The researches that have been made as to a proper solvent for cocain depart from the principle that an injection made in the tissues will be the less dangerous in proportion as its diffusibility is lessened; that is to say that it should enter as tardily and slowly as possible into the circulatory torrent.

We therefore sought to use as a vehicle for the introduction of cocain such substances as oil, vaseline and cocoa-butter. After explaining their mode of use, we arrive at the following conclusions: Such substances used in the transmission of cocain have the disadvantage of determining, by their presence in the tissues, all the accidents attributable to foreign bodies. They sensibly retard cicatrization and many of them produce sphacele. From which we conclude that the use of vehicles other than distilled water appear to us to possess only disadvantages, with no profit whatsoever.

Cocain being a toxic medicament, one should use as small a dose as possible, in spite of the fact of its being endowed with sufficient analgesic power. There is a general agreement to-day to recognize a one percent (1 to 100) solution as sufficient.

The toxicity of cocain depends, not only on the weight of the injected alkaloid, but also on the quantity of water in which it is dissolved. The greater the quantity of water used, the less offensive it is for the same amount of cocain.

For the extraction of a tooth, twenty drops of a 1 to 100 solution, that is to say, one-sixtieth of a grain of cocain, quite suffices.

Schleich employs even a much feebler solution; but in this case, the anesthesia is illusionary and due simply to the mechanical distention of the tissues; similar results can be obtained by a simple injection of distilled water.

Ordinarily for the extraction of a tooth, we use one-sixth of a grain or less of cocain. Under twelve years and above sixty, we reduce the dose to no more than one-half this amount. We have never had an accident and have yet always produced perfect anesthesia. In certain cases of complicated extractions, we inject as high as one-half a grain without accident, but conform, of course, to the precautions stated above.

We make use of fresh extemporaneous solutions, or of solutions preserved in sterilized ampoules.

Twenty drops of a fresh and sterile solution of cocain, one part to a hundred of distilled water, seems necessary and sufficient for practice in the majority of cases, and such a dose induces neither accident nor incident.

Any clothes which might in any way impede respiration; a horizontal position, or at any rate one approaching the horizontal should be adopted. There are counter-indications as cocain raises the blood pressure, it is contra-indicated for aortics and ateriosclerous patients.

Because of its depressive action, it ought also to be forbidden for anemic individuals and those debilitated, extremely nervous neurasthenics and to those worn out with debilitating diseases. Should the patient present a general state which would seem to visibly predispose him to syncope, it were better to abstain. These counter-indications are not absolute, they are but relative, and the more to be observed, as the disease seems to arrive at a more serious degree.

When an injection of cocain has been given for the extraction of a tooth whose pulp is exposed, and if at the very moment of extraction the pulp be touched with a sound, the patient is conscious of the pain of that touch, with no attenuation whatever, whereas the extraction itself is absolutely painless.

Where, however, the alveolo-dental articulation is inflamed, the pain is on the contrary the greater and the more intense, as the arthritis itself is more acute.

Consequently, it must be inferred that the pain of extraction is caused by the tearing of the alveolo-dental ligament as a matter of absolute fact. Therefore, if the anesthesia by cocain is to be effective, it must act on the nerve termini of this ligament.

As the gums offer quite a resistance to the injection, it is well to use a good hypodermic syringe with a needle screwed on. Steel needles, by reason of their rigidity and the delicacy of their vent, are preferable.

A great many of the accidents of infection attributed to the cocain are really due to aseptic faults. We keep our syringes in a five percent solution of carbolic acid with the piston drawn out, so as to keep the body of the pump and the piston-rod constantly in contact with the antiseptic liquid. The needles are to be boiled during at least five minutes, or held in the flame.

The mouth to be rinsed with boricated water and the gums washed with alcohol.

As the first puncture is liable to be somewhat painful, even with fine needles, we apply to the gum previously dried a ten percent solution of cocain.

To insure the efficacy of the injection, it must be made at the level of the mucous membrane, which adheres closely to the periostium and consequently not too near the neck, neither too near the vestibulary cul de sac.

The syringe, armed with its needle and held like a pen, is forced into the gum, not deeply, within the derma, at a point situated about equi-distant between the free edge of the gum and the presumed spot where the point of the root should be, obliquely in reference to the median region of the maxillary. A sufficient resistance is felt when injecting and bit by bit the mucous membrane whitens under the influence of the cocain.

One can be sure that the anesthesia will be excellent if the piston pushes hard. If the liquid enters without resistance, it shows that the injection has been made in the cellular tissue and the formation of an œdemous bulb will be determined. It is better then to withdraw the needle and begin again. Several injections are necessary, at least two, in order to surround the tooth with an anesthetized zone.

Adrenaline added to cocain has given good results, from the local as well as from the general point of view.

Pounded ice and pulverized ether have been used to produce local anesthesia, but these methods have to-day only a historical interest.

After having studied the mechanism of anesthesia by refrigeration, paralysis of the extremities, of the sensitive nerves, and vaso-constriction, after having exposed the physical laws governing the evaporation of liquids, we arrive at a consideration of the chlorid of methyl and of ethyl.

Chlorid of Methyl.—Boiling point, 23 degrees below zero (centigrade), produces therefore an intense cold which makes it difficult to handle. We expose the stypage method and conclude that chlorid of methyl is little used because it frequently causes eschars.

Chlorid of Ethyl.—Boiling point 12 degrees above zero (centigrade.) It is employed by spraying on the gums.

We cannot repeat here the manner of using it. We come to the conclusion that chlori d ofethyl renders real service, but that it can hardly be used except for the front teeth, and that the anesthesia produced by it is fugitive and not at all deep.

Finally, after a lengthy study of the properties, practical value, the mode of employing mixtures of chlorid of methyl and of chlorid of ethyl, or coryl, of anesthyl, we terminate this long chapter with the conclusion that refrigeration carefully carried out constitutes an excellent method of local anesthesia for superficial operations and may be used together with cocain injections to secure profound results.

Stovaine is a substance recently discovered by a French chemist, M. Fourneau, which seems to offer some advantages over cocain.

Weaker toxicity.—From experiments on rabbits and guinea pigs, it is shown that the difference of toxicity between the two substances is very great; in fact, cocain is shown to possess twice the toxicity of stovaine. It possesses a vaso-dilative action, while cocain's action is vaso-constrictive. Consequently, with stovaine, a patient may be operated upon, seated. Stovaine costs very much less than cocain. It is about two months since we began using stovaine, that is to say, about the end of January, 1904, upon the advice of Prof. Reclus. This surgeon has been employing it for several months in his service, and has obtained perfect results up to the present.

Personally, we have made a hundred injections of a 0.75 percent solution of stovaine. We have observed no menace of syncope, no sickness ensues and we can affirm that the anesthesia produced is equal to that produced by cocain.

### CONCLUSIONS.

1. Complete anesthesia, because of the dangers and inconveniences it entails, should be the exception in dental surgery. On this fact is based the importance of local anesthesia.

2. Of all the methods of local anesthesia actually known, cocain seems to give the best practical results.

3. Hydrochlorate of cocain appears to us superior to all the other preparations which compete with it.

4. Distilled water is the best vehicle proposed for the transmission of cocain.

5. In general practice, a satisfactory anesthetic is obtained, and all accident is avoided by the use of not more than twenty drops of a fresh one percent solution of hydrochlorate of cocain in distilled water.

6. When the injection exceeds one-sixth of a grain of cocain, a horizontal position is called for.

7. In the extraction of a tooth, it is almost exclusively the rending of the alveolo-dental ligament that causes the pain.

8. The anesthesia is entirely dependent upon the manner of operating the injection. The after-effects depend on the asepsis.

9. After the operation, the patient should remain reclining one-fourth of an hour for one-sixth of a grain of cocain, from two to three hours for a larger dose.

10. Well-executed refrigeration constitutes a good local anesthetic but only for very superficial operations.

11. The mixed method (cocain injection and refrigeration) constitutes the best local anesthetic.

12. Stovaine, a new product, vaso-dilator, powerful local anesthetic, less toxic than cocaine, has given the best of results up to date.

